# Gene Arrangement Convergence, Diverse Intron Content, and Genetic Code Modifications in Mitochondrial Genomes of Sphaeropleales (Chlorophyta)

**DOI:** 10.1093/gbe/evu172

**Published:** 2014-08-08

**Authors:** Karolina Fučíková, Paul O. Lewis, Diego González-Halphen, Louise A. Lewis

**Affiliations:** ^1^Department of Ecology and Evolutionary Biology, University of Connecticut; ^2^Instituto de Fisiología Celular, Departamento de Genética Molecular Universidad Nacional Autónoma de México, Ciudad de México, Mexico

**Keywords:** collinearity, gene fragmentation, genome rearrangements, green algae, rRNA

## Abstract

The majority of our knowledge about mitochondrial genomes of Viridiplantae comes from land plants, but much less is known about their green algal relatives. In the green algal order Sphaeropleales (Chlorophyta), only one representative mitochondrial genome is currently available—that of *Acutodesmus obliquus*. Our study adds nine completely sequenced and three partially sequenced mitochondrial genomes spanning the phylogenetic diversity of Sphaeropleales. We show not only a size range of 25–53 kb and variation in intron content (0–11) and gene order but also conservation of 13 core respiratory genes and fragmented ribosomal RNA genes. We also report an unusual case of gene arrangement convergence in *Neochloris aquatica*, where the two *rns* fragments were secondarily placed in close proximity. Finally, we report the unprecedented usage of UCG as stop codon in *Pseudomuriella schumacherensis*. In addition, phylogenetic analyses of the mitochondrial protein-coding genes yield a fully resolved, well-supported phylogeny, showing promise for addressing systematic challenges in green algae.

## Introduction

**Fig. 1.—:**
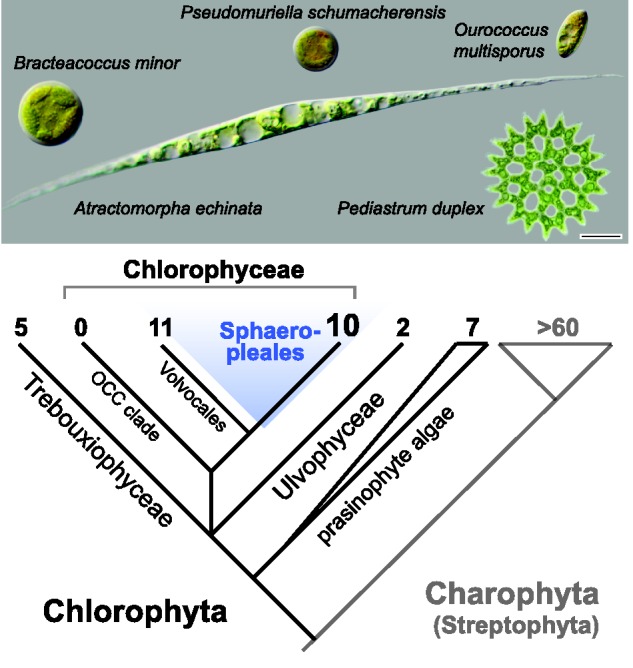
A diagrammatic overview of the diversity of green plants with numbers of species in each lineage for which mt genomes are available. In Sphaeropleales, the number includes one previously published genome and nine genomes newly presented in this study. The micrographs present examples of sphaeroplealean algae examined in this study. Scale bar represents 10 μm.

The mitochondrial (mt) genomes of land plants exhibit great structural and compositional variability (e.g., Palmer and Herbon 1988; Adams et al. 2002; works cited in Adams and Palmer 2003; Knoop 2004). Much less is known about the variation across the remaining Viridiplantae—the diverse green algae of the phyla Chlorophyta and Charophyta (sometimes referred to as Streptophyta, a lineage including all land plants). mt genomes of streptophyte algae recently were investigated in a comparative study by Turmel et al. (2013). Among the Chlorophyta (fig. 1), mt genomes of only a handful of genera have been surveyed. In the class Chlorophyceae, mt genomes from only eight genera have been characterized, and most are limited to the order Volvocales (sometimes referred to as Chlamydomonadales; Denovan-Wright et al. 1998; Kroymann and Zetsche 1998; Smith et al. 2013). mt genome data on the sister group of the Volvocales, the order Sphaeropleales, remain represented only by *Scenedesmus obliquus* (recently recombined as *Acutodesmus obliquus*; Hegewald and Hanagata 2000; mt genome sequenced by Nedelcu et al. 2000). In contrast, chloroplast genomes have been sequenced for representatives of most major lineages across Chlorophyta (e.g., Bélanger et al. 2006; de Cambiaire et al. 2006, 2007; Brouard et al. 2008, 2010).

The mt genome of *Ac. obliquus* is distinct in several aspects when compared with those of volvocalean algae and other Chlorophyta. Its size and gene content are not nearly as reduced as in the Volvocales, but it still lacks all ribosomal protein genes, the 5S ribosomal RNA (rRNA) gene, and several transfer RNA (tRNA) genes, including for example all four Threonine tRNAs. Additionally, the genes encoding the small and large ribosomal subunits (*rns, rnl*) are fragmented and the fragments scattered across the genome, but not to the extent found in Volvocales. For this reason, the *Acutodesmus* mt genome was considered an “intermediate” between the more orthodox Chlorophyta genome (e.g., Trebouxiophyceae, prasinophyte algae) and the highly reduced and derived Volvocales genome (Nedelcu et al. 2000). Moreover, although the Chlorophyceae use a nonstandard genetic code in their mitochondria by the use of UAG to code for Leucine or Alanine (instead of as a stop codon; Hayashi-Ishimaru et al. 1996), *Acutodesmus* uniquely uses UCA as a stop codon (Kück et al. 2000).

The “intermediate” character of the *Acutodesmus* mt genome is well exemplified by the evolutionary history of the *cox2* gene. It was previously noted that prasinophytes and trebouxiophytes have an intact *cox2* gene in their mitochondria. In Volvocales the *cox2* gene can be found in the nuclear DNA as two fragments, *cox2a* and *cox2b* (Pérez-Martínez et al. 2001; Prochnik et al. 2010), which presumably migrated there from the mitochondrion independently of one another. *Acutodesmus* has the *cox2a* gene in the mitochondrion (Nedelcu et al. 2000), suggesting that mt *cox2* was fragmented and *cox2b* transferred to the nucleus first, in the ancestor of Chlorophyceae, and *cox2a* migrated to the nucleus later, in the ancestor of Volvocales. This hypothesis was recently tested and supported using targeted sequencing on an expanded sampling of species across the class Chlorophyceae (Rodríguez-Salinas et al. 2012). Although colonial forms such as *Acutodesmus* often are used to represent Sphaeropleales, the majority of the order’s 16 families comprises solely or predominantly simple spherical unicells (Fučíková et al. 2014). This study greatly expands the sampling of the mt genome diversity within Sphaeropleales by presenting nine new, complete mt genomes spanning the majority of the order, and by contributing mt gene sequences from three additional members of Sphaeropleales. We test whether the *Acutodesmus* mt genome is representative of the entire order and whether the mt genome diversity in Sphaeropleales is comparable to its sister order, Volvocales. More specifically, we address the following questions: 1) Are the same genes present/absent across Sphaeropleales? 2) Is the configuration of the *cox2* gene consistent with the findings of Rodríguez-Salinas et al. (2012)? 3) Are the rRNA genes always fragmented and scrambled in the same manner? 4) Is the same genetic code used across the order? Additionally, we examined the potential phylogenetic utility of the protein-coding genes and the structural characteristics of the mt genomes.

## Materials and Methods

Algal strains were obtained from the Culture Collection of Algae at the University of Texas at Austin (UTEX, http://www.utex.org, last accessed August 13, 2014), the Culture Collection of Algae at the University of Göttingen, Germany (SAG, http://sagdb.uni-goettingen.de, last accessed August 13, 2014), and the Culture Collection of Algae of Charles University in Prague (CAUP, http://botany.natur.cuni.cz/algo/caup-list.html, last accessed August 13, 2014) and were maintained under 16:8 light:dark cycle at 18 ° C and 70 µmol m^−^^2 ^s^−^^1^ in liquid media or on agar slants as specified by the institution of origin.

Genomic DNA was extracted using a PowerPlant DNA Isolation Kit (MO-BIO Laboratories, Carlsbad, CA), with a modified purification part of the protocol using chloroform separation and ethanol precipitation steps instead of column cleaning. DNA was subsequently shipped to Cold Spring Harbor Laboratories for TruSeq library preparation and sequencing on Illumina HiSeq2500, producing 2 × 100 bp paired reads. The reads were paired, trimmed, assembled, and annotated in Geneious v.R6 (Biomatters; www.geneious.com). For the large data sets of *Bracteacoccus minor* (46 million reads) and *Neochloris aquatica* (33 million reads), the program ABySS (Simpson et al. 2009) was used for initial assembly. In most cases, a single mt contig was obtained from de novo assembly. In cases where multiple contigs were obtained (e.g., in *Chromochloris zofingiensis*), the contigs were subjected to a series of reference assemblies in Geneious (by mapping reads to the mt fragments and subsequently to the longer resulting fragments, often for 25 iterations or more) until we reached a point where the fragments could be confidently joined. The final contigs were verified by mapping paired reads to the consensus sequences and inspecting the resulting assemblies by eye for mismatches or unexpected drops in coverage. The coverage of the mt genomes varied from 40× to 1,800×. Large regions with tandem repeats were identified from spikes in coverage, verified by inspection by eye, and indicated in the National Center for Biotechnology Information submissions as regions with an uncertain number of repeats. Smaller tandems were identified using a perfect search for 8–30 nt units in Phobos (Mayer 2006–2010) and dispersed repeats were identified using RepeatMasker (Smit et al. 1996–2010).

Annotation was aided by the combination of the following tools: DOGMA (Wyman et al. 2004; dogma.ccbb.utexas.edu/, last accessed August 1 2014), RNAweasel (Lang et al. 2007; http://megasun.bch.umontreal.ca/cgi-bin/RNAweasel/RNAweaselInterface.pl, last accessed August 1, 2014), and BLAST (http://blast.ncbi.nlm.nih.gov/, last accessed August 13, 2014). Intron boundaries were determined based on alignments of the exon amino acid and nucleotide sequences. Intron-conserved secondary structure elements were identified by RNAweasel, which also determined the intron subgroup affiliation. Intronic open reading frames (ORFs) were predicted in Geneious and the characteristic intronic domains were detected using the BLASTx tool (http://ncbi.nlm.nih.gov/, last accessed August 13, 2014). The final annotations including exon boundaries were checked using manual alignments with data from published chlorophycean mt genomes. Codon usage was analyzed in Geneious and synteny maps were produced using the Mauve plugin in Geneious, which was also used to estimate the numbers of genomic rearrangements (Darling et al. 2004). Genome map figures were prepared using OGDRAW (Lohse et al. 2007) and edited in Adobe Illustrator. Sidedness index (*C*_S_) was calculated according to Cui et al. (2006).

Individual mt genes were aligned using the Geneious translational alignment and subsequent manual adjustments, and unalignable codons were identified and removed manually prior to phylogenetic analyses. In addition to genes from taxa with completed mt genome sequences, protein-coding gene sequences were obtained from partially sequenced genomes of *Atractomorpha echinata* (UTEX 2309, GenBank accession numbers KJ845680–KJ845692), *Rotundella rotunda* (UTEX 2979, GenBank accession numbers KJ845706–KJ845718), and *Pediastrum duplex* var. *asperum* (UTEX LB1364, GenBank accession numbers KJ845693–KJ845705). The published sequences of *Ac. obliquus* (UTEX 78, AF204057) and *Stigeoclonium helveticum* (UTEX B441, EU123950–EU123956) were also used in phylogenetic analyses. Volvocalean outgroup taxa were not included because Volvocales have lost an additional six mt protein-coding genes and transferred them to the nucleus (Smith et al. 2013), and the remaining seven are very divergent from all other chlorophyceans, often truncated and difficult to align.

The concatenated 13-gene data set was partitioned into five subsets as proposed by PartitionFinder (Lanfear et al. 2012), and analyzed using Garli v.2.0 (Zwickl 2006) under the GTR+I+G model of evolution for each subset. Five independent searches for the best tree were conducted in each analysis. A corresponding Bayesian analysis using Phycas (Lewis et al. 2011) was conducted on the partitioned data set, carrying out 100,000 cycles with polytomies allowed, sampling every 100 cycles. The first 200 samples of the run were discarded as burn-in. Bayesian marginal posterior probabilities (BPP) of splits were estimated from the postburn-in sample of trees. Comparable analyses were conducted on the nuclear rDNA and four-gene chloroplast data sets similar to those in Fučíková et al. (2014) using the same set of taxa as the present study in order to directly compare the phylogenetic signal from mt genes to that of chloroplast and nuclear ribosomal genes (18S, 5.8S, and 28S). The four-gene chloroplast data set (including *psaB*, *psbC*, *rbcL*, and *tufA*) was partitioned by codon position. Additionally, we analyzed all of the available data (nuclear, chloroplast, and mt) in combined maximum likelihood (ML) and Bayesian analyses, partitioned according to PartitionFinder (Lanfear et al. 2012) into ten subsets.

We also examined the phylogenetic utility of gene order using BADGER (Simon and Larget 2004). Because BADGER does not consider gene loss and because not all tRNA genes were present in all of our taxa of interest, we only included 36 genes that were common to all ten taxa with completely sequenced genomes. Two runs of 5,000,000 cycles were performed, sampling every 100 cycles and the first 200 samples of each run were discarded as burn-in.

## Results

### Genome Size and Content

**Fig. 2.—:**
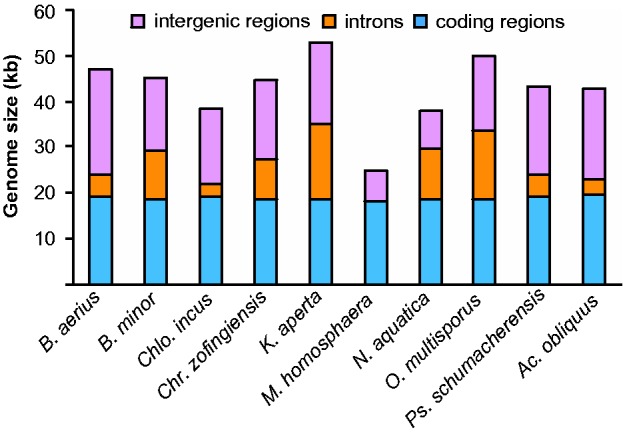
Comparison of mt genome content across Sphaeropleales with respect to proportions of coding DNA (respiratory protein-coding genes, tRNA genes, and rRNA genes), intronic DNA, and intergenic DNA. *Acutodesmus obliquus* is a synonym of *Scenedesmus obliquus*.

The mt genomes of the surveyed Sphaeropleales ranged greatly in size and content (supplementary figs. S1–S9, Supplementary Material online), but in general, 13 respiratory protein-coding genes (including *cox2a* but not *cox2b*), 22–27 tRNA genes, 4 fragments of *rnl*, and 2 fragments of *rns* were present. The overview of the characteristics of the genomes is presented in table 1, and the proportions of coding versus intronic versus intergenic DNA are presented in figure 2. Information on presence/absence of tRNA genes is presented in supplementary table S1, Supplementary Material online. The *cox2a* gene was present and the *cox2b* gene was absent in all completed and partial genomes examined. Several taxa had one or more regions of tandem repeats, in particular, two large stretches of tandem repeats were found in *Kirchneriella aperta*: One 26-mer repeated at least ten times starting at position 21672, and one 27-mer repeated at least ten times starting at position 51906. Searches for dispersed repeats found several AT-rich repetitive elements between 6 and 10 nucleotides in length. Only two of these elements were found in more than one taxon: TTTTGG in *Chlorotetraedron* and *Pseudomuriella*, and TTAATT in *Neochloris* and *Ourococcus* (supplementary table S2, Supplementary Material online). None of the repeats resembled those described in Nedelcu and Lee (1998).

**Table 1:** **Summary of Characteristics of Nine Newly Sequenced** mt **Genomes from the Order Sphaeropleales, with the Previously Published mt Genome of *Acutodesmus obliquus* (as *Scenedesmus obliquus*)**

Species	Strain Number	GenBank Accession No.	Size (kb)	% GC	% Coding	%GC in Coding	Group I Introns	Group II Introns	Intronic ORFs*	tRNAs	No. Sided Blocks
*Bracteacoccus aerius*	UTEX 1250	KJ806265	47.2	47.1	40.8	50.6	2	1	4	24	4
*Bracteacoccus minor*	UTEX B66	KJ806266	45.2	43.0	42.2	48.9	5	1	7	24	4
*Chlorotetraedron incus*	SAG 43.81	KJ806267	38.4	37.7	49.7	42.2	2	0	3	24	2
*Chromochloris zofingiensis*	UTEX 56	KJ806268	44.8	35.0	42.3	40.9	6	3	4	24	1
*Kirchneriella aperta*	SAG 2004	KJ806269	52.9	37.1	36.2	42.8	6	3	5	25	2
*Mychonastes homosphaera*	CAUP H6502	KJ806270	25.1	43.0	72.8	44.9	0	0	0	22	4
*Neochloris aquatica*	UTEX 138	KJ806271	38.0	34.4	49.2	39.4	11	0	3	24	2
*Ourococcus multisporus*	UTEX 1240	KJ806272	49.7	32.1	38.1	40.8	9	1	7	22	2
*Pseudomuriella schumacherensis*	SAG 2137	KJ806273	43.1	42.8	44.4	47.7	4	0	3	26	6
*Acutodesmus obliquus*	UTEX 78	AF204057	42.9	36.3	45.7	40.7	2	2	1	27	2

The smallest genome was that of *Mychonastes homosphaera*, 25.1 kb, which was also completely devoid of introns. The largest genome was that of *K. aperta* at 52.9 kb, and the most introns (11) were found in *N. aquatica*. The fewest sided blocks (determined according to Cui et al. 2006) were present in *Chr. zofingiensis*, where all genes were found on the same strand (*C*_S_ = 1), and the most (6) in *Pseudomuriella schumacherensis* (*C*_S_ = 0.89).

### Intron Content and Diversity

**Fig. 3.—:**
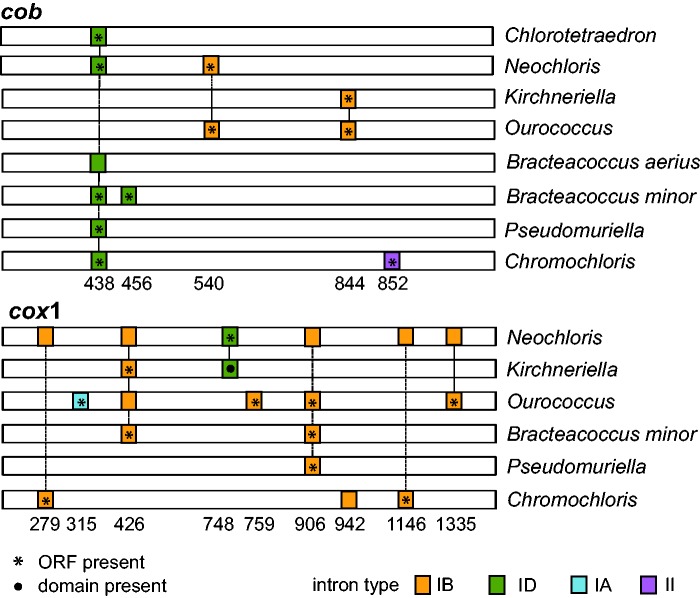
Overview of intron abundance and diversity in two respiratory mt genes of Sphaeropleales, *cob* and *cox1*. Introns inserted at corresponding positions in each gene are connected with vertical lines. Positions in each gene are counted from the first nucleotide of the start codon of *Bracteacoccus aerius*. Presence of uninterrupted intronic ORFs is indicated with asterisks (*) and the presence of recognizable intronic protein domains is indicated with a circle.

The most common introns were the group IB and often contained one or two recognizable LAGLIDADG domains in an uninterrupted ORF. Less frequent were group IA, ID, and group II introns. Other types of intronic ORFs were occasionally detected, including GIY–YIG domains, or multiple domains in a single intron generally including reverse transcriptase, maturase, and HNH domain (this type is most commonly associated with group II introns). In Sphaeropleales, introns were commonly found in *rnl*4, *cob*, and *cox1*; rarely in *nad5* (*Acutodesmus*), *rns1* (*Acutodesmus*), and *rnl2* (*Kirchneriella*). The distribution and diversity of introns are exemplified in figure 3 using the information from *cob* and *cox1*. The *rnl4* gene often contained one or two group II introns (six taxa; only completed genomes considered), and almost always one or two group IB introns (nine taxa), generally with a putative LAGLIDADG homing endonuclease ORF, and occasionally one or two additional group IA introns (5 taxa). In both *Bracteacoccus* species, putative ORFs with characteristic intronic domains (LAGLIDADG in both species and GIY–YIG in *B. aerius*) were detected in intergenic spacers. In the *cob* gene of *Chlorotetraedron incus,* two putatively unrelated ORFs were detected with the structural group ID element between them. In addition, in some taxa intronic domains were recognized within intron regions, but a corresponding uninterrupted ORF was not detected. All introns, their conserved secondary structure elements, recognizable internal ORFs, and characteristic protein domains (associated with ORFs or not) are annotated in the GenBank accession numbers.

### Genomic and Genic Rearrangements

**Fig. 4.—:**
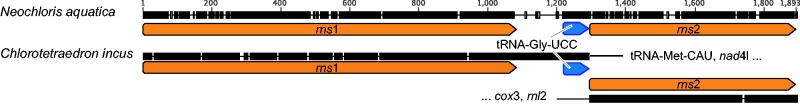
Geneious alignment of the mt small ribosomal subunit (*rns*) gene of *Neochloris aquatica*, where the two fragments, *rns1* and *rns2*, are in close proximity, and *Chlorotetraedron incus*, where the fragments are in different parts of the genome. In both taxa, *rns1* is adjacent to tRNA gene for Glycine (UCC).

Unlike any other pair of taxa we examined, *K. aperta* and *O.multisporus* had completely collinear mt genomes, that is, an identical gene order. All other pairs of taxa were separated by multiple genomic rearrangements (estimated using double-cut-and-join (DCJ) analysis of the Mauve plugin in Geneious). The *rns* gene was present in two fragments, *rns1* and *rns2*. In the *Neochloris* genome, RNAweasel detected a single *rns* gene, but upon further examination separate *rns1* and *rns2* genes were recognized, with a tRNA-Gly (UCC) gene between them (fig. 4). The *rnl* gene was consistently present in four fragments scattered across the genomes.

Gene order phylogenetic analysis using BADGER (Simon and Larget 2004) yielded a tree with good support for groupings of *Ourococcus* + *Kirchneriella*, *Bracteacoccus aerius* + *B. minor*, *Neochloris* + *Chlorotetraedron*, and *Ourococcus* + *Kirchneriella* + *Neochloris* + *Chlorotetraedron* + *Acutodesmus* (supplementary fig. S11, Supplementary Material online). Other nodes in the tree received moderate to low support.

### Genetic Code and Codon Usage

Most mt genes of the examined sphaeroplealean algae translated well under the *Acutodesmus* mt code. Most taxa appeared to use both UCA and UAA as stop codons, except for *K. aperta* and *Ac. obliquus*, which only used UCA, and *Ps. schumacherensis*, which only uses UCA and, in four cases (*nad2*, *nad3*, *nad4L*, and *cox1*) UCG, which is unprecedented to our knowledge. UCG is not used as a sense codon in any of the sphaeroplealean mt genes, except in one instance in the *cox*3 gene of *Chromochloris*, where it could be a plausible stop codon. The corresponding tRNA (normally coding for Serine) is absent in all the genomes examined here. The data from the partially assembled genomes of *At. echinata* and *R. rotunda* suggested an UAG stop codon in the *nad1* gene (table 2).

**Table 2:** **Stop Codon Usage in** mt **Respiratory Genes across Sphaeropleales, Including Data from Nine Newly Obtained mt Genomes, Three Partially Sequenced Genomes (*Atractomorpha echinata*, *Pediastrum duplex*, and *Rotundella rotunda*), and the Previously Published Genome of *Acutodesmus obliquus* (as *Scenedesmus obliquus*)**

Taxon	*nad*1	*nad*2	*nad*3	*nad*4	*nad*5	*nad*6	*nad*4L	*cox*1	*cox*2a	*cox*3	*cob*	*atp*6	*atp*9
*Bracteacoccus aerius*	UCA	UCA	UCA	UCA	UCA	UCA	UCA	UAA	UAA	UCA	UAA	UAA	UAA
*Bracteacoccus minor*	UCA	UCA	UCA	UCA	UCA	UCA	UCA	UCA	UCA	UCA	UCA	UCA	UAA
*Chlorotetraedron incus*	UAA	UAA	UAA	UAA	UCA	UAA	UAA	UCA	UCA	UAA	UCA	UCA	UAA
*Chromochloris zofingiensis*	UCA	UCA	UAA	UCA	UAA	UCA	UAA	UAA	UCA	UCA	UAA	UAA	UAA
*Kirchneriella aperta*	UCA	UCA	UCA	UCA	UCA	UCA	UCA	UCA	UCA	UCA	UCA	UCA	UCA
*Mychonastes homosphaera*	UCA	UAA	UCA	UCA	UCA	UCA	UCA	UCA	UCA	UAA	UCA	UCA	UCA
*Neochloris aquatica*	UCA	UCA	UCA	UAA	UCA	UAA	UAA	UAA	UAA	UAA	UCA	UCA	UCA
*Ourococcus multisporus*	UCA	UCA	UCA	UCA	UAA	UAA	UCA	UCA	UCA	UCA	UAA	UAA	UAA
*Pseudomuriella schumacherensis*	UCA	UCG	UCG	UCA	UCA	UCA	UCG	UCG	UCA	UCA	UCA	UCA	UCA
*Acutodesmus obliquus*	UCA	UCA	UCA	UCA	UCA	UCA	UCA	UCA	UCA	UCA	UCA	UCA	UCA
*Pediastrum duplex*	UCA	UAA	UAA	UAA	UAA	UAA	UAA	UAA	UAA	UAA	UAA	UAA	UAA
*Rotundella rotunda*	UAG	UCA	UAA	UCA	UAA	UCA	UAA	UCA	UCA	UCA	UAA	UCA	UCA
*Atractomorpha echinata*	UAG	UAA	UAA	UAA	?	?	UAA	UAA	UAA	UAA	?	UAA	UAA

Note.—Question marks (?) indicate incomplete/unavailable data.

### Phylogenetic Analyses

**Fig. 5.—:**
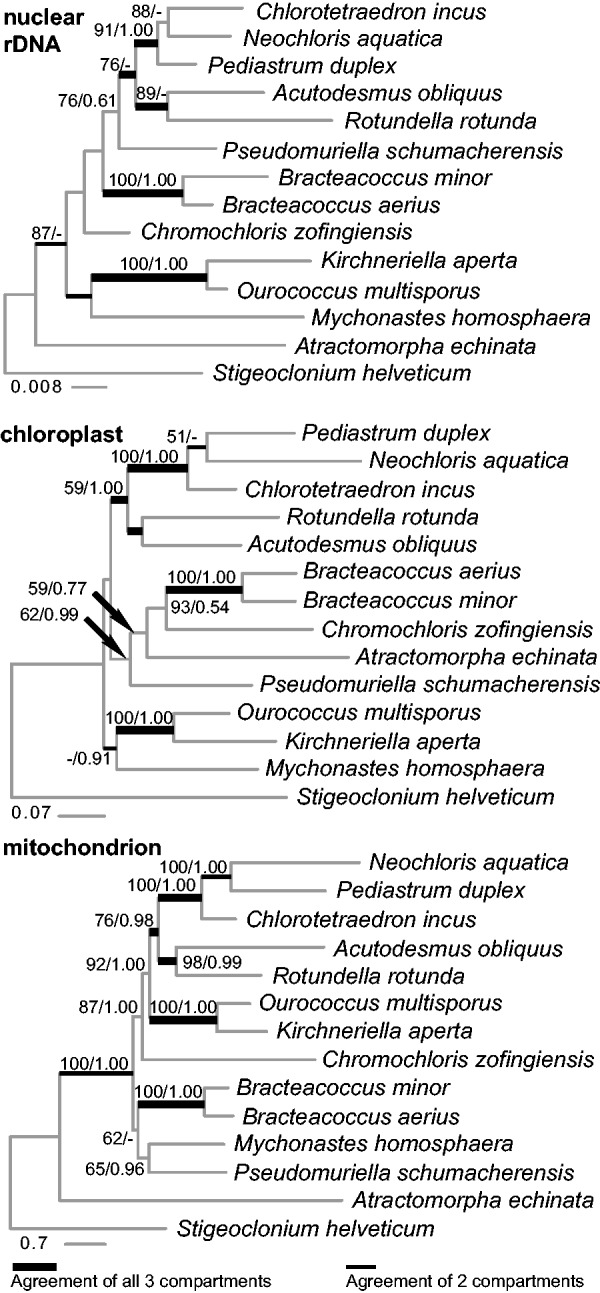
Comparison of phylogenies yielded by ML analyses of nuclear rDNA genes, four chloroplast genes, and 13 mt genes. Numbers at branches represent ML bootstrap (BS) support values and BPP, respectively. Dashes (-) indicate support lower than 50 BS or 0.50 BPP, respectively. Scale bars represent estimated numbers of substitutions per site. *Acutodesmus obliquus* is a synonym of *Scenedesmus obliquus*.

The concatenated alignment comprised 14 taxa and 10,728 sites from the 13 core respiratory genes after removal of 2,466 nucleotides (or 822 codons) of uncertain homology. The only case of missing data was *atp*6 of the partially published genome of *S. helveticum*. The selected partitioning scheme was as follows: (*atp6*, *cox3*, *nad2*, *nad4*, and *nad5* first codon positions), (*atp*6, *cox3*, *nad2*, *nad4*, and *nad5* second codon positions), (all third codon positions), (*atp9*, *cob*, *cox1*, *cox2a*, *nad1*, *nad3*, *nad4L*, *nad6* first positions), and (*atp*9, *cob*, *cox1*, *cox2a*, *nad1*, *nad3*, *nad4L*, *nad6* second positions). The Bayesian and ML analyses yielded near-identical topologies and most nodes received moderate to good support from both types of analysis (fig. 5). Only the clade grouping the two *Bracteacoccus* species with *Mychonastes* and *Pseudomuriella* received low ML support and was not recovered in the Bayesian analysis—instead, in the Bayesian analysis *Bracteacoccus* was placed as sister group to all other Sphaeropleales except the deeply diverging *Atractomorpha*.

For the same 14 taxa, a data set of four chloroplast genes and a data set of nuclear rRNA were analyzed in order to compare the phylogenetic signal from the three cellular compartments (mt, cp, and nu). The analyses of chloroplast genes and nuclear rRNA genes yielded results largely consistent with Fučíková et al. (2014). The comparison of the chloroplast, mt, and nuclear phylogenies is presented in figure 5. Results of all three analyses were in agreement for the grouping *B. aerius* with *B. minor*, *Pe. duplex* with *Chlo. incus* and *N. aquatica*, *Ac. obliquus* with *R. rotunda*, the five latter taxa together, and *O. multisporus* with *K. aperta*. The chloroplast and mt phylogenies agreed on the grouping of *Neochloris* with *Pediastrum*, with *Chlorotetraedron* being sister to them, and the mt and nuclear phylogenies agreed on placing *Atractomorpha* as sister to the remaining Sphaeropleales. The nuclear and chloroplast data supported the grouping of *Kirchneriella* and *Ourococcus* with *Mychonastes*.

The analyses of all data combined yielded a topology that was identical to that based on mt data only (fig. 5 and supplementary fig. S10, Supplementary Material online). Most nodes in these combined analyses received good support that was either as high as or higher than in the mt data analyses. The only exception was the placement of *Chromochloris*, which received lower support than in the mt analyses.

## Discussion

Organellar genomes, especially mt genomes, remain largely uncharacterized in chlorophyte algae (e.g., Bélanger et al. 2006; de Cambiaire et al. 2007; Brouard et al. 2008, 2010—chloroplast genome completed but not mt). In the class Chlorophyceae, the bulk of the information about mt genomes is concentrated in the order Volvocales, whereas only one nonvolvocalean taxon has been surveyed: *Acutodesmus*. Given the variation in size, architecture, and content found in green algal chloroplast and mt genomes so far, we can only expect to find more diversity as we sample more densely within the major groups.

Land plant mt genomes are known for their great size variation, horizontal gene transfer, and frequent rearrangements (e.g., Palmer and Herbon 1988; Adams et al. 2002; Mach 2011). Their coding regions generally maintain their integrity, although sometimes they are transferred to the nucleus. In this study, sampling across Sphaeropleales revealed considerable diversity including features unique within green algae, but the size and structural complexity do not approach the extremes found in land plants. Analogously to land plants, however, the coding sequences mostly appeared intact (although sometimes interrupted by introns), and contained phylogenetic signals comparable to commonly used chloroplast markers.

Volvocales can be viewed as the opposite extreme to land plants. They are known for their highly reduced mt genomes, which generally only contain seven protein-coding genes, highly fragmented rRNA genes, and two to three tRNA genes. However, although overall their mt genomes are smaller than those of most green plants, there is great variation across the taxa sampled thus far. On one end of the volvocalean spectrum is *Polytomella capuana* with its linear mt genome high in GC and the smallest known among Archaeplastida (Smith and Lee 2007). On the other end is *Volvox carteri*, whose mt genome is approximately 30 kb in size (large for Volvocales but still smaller than most green plants) and bloated with over 60% noncoding DNA (Smith and Lee 2009). In addition to the common circular structure of the mt genomes and the linear arrangement in, for example, *Po. capuana* and *Chlamydomonas reinhardtii*, some Volvocales have been demonstrated to have a fragmented-linear arrangement of mt genomes (Smith et al. 2010). Compared with this variability, Sphaeroplealean mt genomes are fairly conserved.

### Genome Size and Content

As far as our first research question is concerned, the mt genome of *Ac. obliquus* indeed appears to be representative of Sphaeropleales in many ways. Its size, GC content, and percentage of coding sequence are well within the order’s range. Like all other Sphaeropleales examined, *Acutodesmus* has 13 respiratory genes in its mt genome, 2 *rns* fragments and 4 *rnl* fragments, and uses a nonstandard genetic code. Nonrepresentative features of *Acutodesmus* among Sphaeropleales include introns in *rns1* and *nad1* genes, as well as the lack of use of UAA as stop codon (also seen in *Kirchneriella*).

The smallest sphaeroplealean mt genome was that of *M. homosphaera*, comprising 25,149 bp, lacking introns completely, and containing very little noncoding sequence (72.7% coding sequence, highest among Sphaeropleales). This puts *Mychonastes* in the size range of volvocalean mt genomes, whereas its genome contains all genes characteristic for Sphaeropleales. A recent study by Fučíková et al. (2014) placed *Mychonastes* as the deepest diverging member of Sphaeropleales based on an analysis of four chloroplast genes. However, although the overall character of the mt genome might support this hypothesis, the phylogenetic information coming from sequences of the mt genes favors the placement of *Mychonastes* well within Sphaeropleales, with *Atractomorpha* being the deepest diverging member of the order, consistent with analyses of nuclear ribosomal genes (Keller et al 2008; Fučíková et al. 2014).

### Intron Content and Diversity

Intron diversity and distribution in the examined genomes are also remarkable. Certain genes (*cob*, *cox1*, and *rnl4* especially) appear prone to intron invasion, often at the same positions, but the homology of these introns is uncertain and the insertion could happen independently in different taxa. None of the introns determined in this study could be interpreted as ancestral and transmitted vertically throughout the order. In fact, even between close relatives (*B. aerius* vs. *B. minor*, *K. aperta* vs. *O. multisporus*) differences in intron content are found, although some introns may be shared (*cob* and *cox1* examples shown in fig. 3). This is analogous to the findings of McManus et al. (2012) and further indicates that intron presence/absence may not be a good predictor of deep phylogenetic relationships, especially when taxon sampling is sparse.

No intron could be confidently identified as both widespread in and unique to Sphaeropleales. Most of the *cob* and *cox1* introns also occur in other Chlorophyta. The best example is the group IB intron at position 1146 in *cox1*, also found in the volvocaleans *Chlamydomonas eugametos*, *Pleodorina starii*, and *Volvox carteri*, the ulvophycean *Pseudendoclonium akinetum*, and the prasinophyte *Monomastix* sp. It is unclear whether this is a case of multiple losses or multiple acquisitions of the intron.

### Genomic and Genic Rearrangements

Mauve alignments show that even between close relatives, for example, the congeners *B. aerius* and *B. minor*, several architectural rearrangements are found. This is the case across the entire order, with the exception of *Kirchneriella* and *Ourococcus* (members of the family Selenastraceae), whose genomes are perfectly collinear. The only differences between the two selenastracean genomes (aside from primary sequence differences) appear to be three missing tRNA genes in *Ourococcus* (supplementary table S1, Supplementary Material online). Conserved gene order (collinearity) in plant mt genomes is unusual but not unheard of. Similar, perhaps even more striking, cases of collinearity are found in mosses (spanning true mosses) and liverworts (spanning all liverworts; Liu et al. 2011). *Ourococcus* and *Kirchneriella* are not each other’s closest relatives, and even though the phylogenetic relationships within Selenastraceae are not yet well resolved (Fawley et al. 2005), it is possible that related genera share the same gene order. Additional taxon sampling in the family Selenastraceae will be necessary to determine the phylogenetic depth of this genomic conservation.

Architectural rearrangements of genomes are thought to be correlated with the occurrence of short repetitive sequences (SRS; Nedelcu and Lee 1998), and this hypothesis was specifically proposed and examined with respect to the fragmentation of rRNA genes. Such GC-rich SRS often occur in the flanking regions of rRNA fragments in Volvocales but not Sphaeropleales, and consequently the volvocalean rRNA genes are fragmented and scrambled to a much higher degree. Our data corroborate the model proposed by Nedelcu and Lee (1998), as no evidence of further fragmentation was found within Sphaeropleales, and no obvious GC-rich SRS occur in the taxa examined here.

An unexpected arrangement of the *rns* genes was found in *N. aquatica*. Unlike all other taxa examined, in which the two *rns* fragments are often widely separated and sometimes placed on opposite strands, the mt genome of *N. aquatica* has *rns1* and *rns2* arranged in each other’s close proximity, superficially recognizable as a single *rns* gene (e.g., by RNAweasel). Upon closer examination, however, it becomes clear that an approximately 100-bp region separates the two fragments. Moreover, the tRNA-Gly (UCC) occurs in this region between *rns1* and *rns2*. The same tRNA gene follows the *rns1* of *Chlorotetraedron*, the closest relative of *Neochloris* examined in this study. The interpretation of this arrangement is not straightforward, but the tRNA-Gly (UCC) can be considered a footprint of past separation of the *rns* fragments. It is fair to assume, given the similar character of the rRNA gene fragments across Sphaeropleales, that the fragmentation of *rns* occurred once in the ancestor of the order. It is also unlikely that such fragments subsequently could be placed back in their original, “orthodox” configuration by mere chance. Horizontal transfer is not a likely alternative explanation in our case, as phylogenetic analyses of the conserved regions of *rns* (*rns1* and *rns2* analyzed separately; results not shown) still yield the expected relationship of *Neochloris* and *Chlorotetraedron*. Upon examination of the gene order in Sphaeropleales, the gene cluster *nad3, cox3, rnl2,* and *rns2*, which is conserved in *Pseudomuriella, Mychonastes, Ourococcus, Kirchneriella*, and *Chlorotetraedron* (and parts of it in other taxa as well), appears interrupted in *Neochloris*. In *Neochloris*, a fragment including *atp6, rns1,* and tRNA-Gly (UCC), appears to have been inserted between *rnl2* and *rns2*, resulting in an orthodox-like sequence of *rns1,* tRNA-Gly (UCC), and *rns*2*.* Examination of related species within Neochloridaceae might help illuminate this problem, but with the data at hand it indeed appears that the two *rns* fragments were placed in each other’s proximity secondarily. It is worth noting that electrophoretic results of Nedelcu (1997) suggest that the two *rns* fragments are transcribed separately in *N. aquatica* despite their proximity.

The model proposed for the fragmentation of *cox2* in the evolutionary history of chlorophyte algae (Nedelcu et al. 2000; Rodríguez-Salinas et al. 2012) is also supported by our data. All Sphaeropleales examined have *cox2a* in the mitochondrion and are missing *cox2b*, which is present in the nucleus (Rodríguez-Salinas et al. 2012). Further light can be shed on this evolutionary story by sampling some of the deeply diverging volvocaleans, in order to determine at what point the *cox2a* gene was transferred to the nucleus. Similarly, surveying deep lineages and “incertae sedis” taxa that may have diverged early in the history of Chlorophyceae might reveal the missing link between the fragmentation of the mt *cox2* and the transfer of *cox2b* to the nucleus.

Finally, it might be tempting to consider the *Mychonastes* genome a proxy for the ancestral mt genome for Sphaeropleales—it contains no introns and is small and compact, much like the mt genomes of Volvocales—and it is not difficult to imagine how the remaining sphaeroplealean genomes may have evolved from such a state by acquisition of introns, expansion of intergenic regions, and structural rearrangements. Yet, multiple lines of evidence now suggest that *Mychonastes* is not in fact the deepest diverging sphaeroplealean (Keller et al 2008; Fučíková et al. 2014; this study). In the light of this phylogenetic information, it is still possible that *Mychonastes* represents an ancestral-like form, but the interpretation becomes much less straightforward. The completion of genomes from members of the family Sphaeropleaceae (e.g., *Atractomorpha*) will help elucidate this question.

### Genetic Code

One of the most surprising findings in our study was the variation in the genetic code across Sphaeropleales. The known “Chlorophycean mitochondrial” code was demonstrated by Hayashi-Ishimaru et al. (1996) in three Hydrodictyaceae (family in Sphaeropleales here represented by the data of *Pe. duplex*), where based on alignments of *cox1* sequences UAG was inferred to code for Alanine, and two Scenedesmaceae (here represented by *Ac. obliquus*), where UAG codes for Leucine. The latter was corroborated by the analysis of the whole mt genome of *Ac. obliquus* (Kück et al. 2000). Our study contributes new information about the evolution of genetic code in Sphaeropleales: The UAG sense codon is not used except in *Ac. obliquus* and *N. aquatica*, both of which have the corresponding tRNA in their mt genomes, and occurs once in *nad5* of *O. multisporus*, which does not have the corresponding tRNA in its mt genome. Our partial genome data from *Pe. duplex* indicate common usage of the UAG codon as well as the presence of a Leucine-type tRNA with the anticodon CUA (supplementary table S1, Supplementary Material online). This contrasts with the findings of Hayashi-Ishimaru et al. (1996), who concluded that UAG codes for Alanine in three other hydrodictyacean species, considering the amino acid most common in other plants and algae at the UAG positions as the most likely to be coded for by UAG. In light of our data it seems more likely that Hydrodictyaceae use UAG for Leucine, not Alanine. Our partial data also indicate a single occurrence of UAG in a plausible stop codon position in the *nad1* gene of the deepest diverging Sphaeroplealean, *At. echinata*, and of *R. rotunda* (table 2).

Kück et al. (2000) demonstrated that UCA is a stop codon in *Ac. obliquus*. Our study shows that the UCA stop codon is common to all Sphaeropleales for which we have complete mt genomic data (table 2). Although most protein-coding genes translated well under the *Acutodesmus* code as expected, exceptions were encountered. Most prominent is the usage of UCG as stop codon in *Pseudomuriella*, which to our knowledge has never been reported. It is also noteworthy that stop codon usage differs quite dramatically between closely related taxa (e.g., *B. aerius* vs. *B. minor*; table 2). The *atp9* gene of *Pseudomuriella* also appears to start with a GUG instead of the usual AUG codon. These observed codon modifications are in accordance with the proposal put forward by Nedelcu et al. (2000) that Sphaeropleales retained more genes in their mt genomes than Volvocales due to incompatibilities in their genetic code. This incompatibility restricted further migration of mt genes to the nucleus in sphaeroplealean algae, whereas in their sister order Volvocales *atp6*, *cox2a*, *cox3*, *nad3*, and *nad4L* are now nucleus-encoded.

### Phylogenetic Analyses

The mt phylogeny is well supported at nearly all nodes, whereas in the nuclear and chloroplast phylogenies, deep divergences are poorly supported (fig. 5), and the chloroplast and nuclear topologies are congruent with Fučíková et al. (2014, supplementary figures). The mt phylogeny strongly supports a relationship heretofore unconsidered: The clade grouping *Neochloris, Pediastrum, Chlorotetraedron, Acutodesmus, Rotundella, Ourococcus*, and *Kirchneriella*. These genera represent all of the colony-forming families in Sphaeropleales, suggesting a single origin of the colonial life style (with possible reversals to solitary forms). Our fully resolved, mostly well-supported phylogeny shows that the mt genes hold a lot of promise for resolving deep relationships of green algae. It is also possible that the use of a comparable number of chloroplast or nuclear genes (here we used 4 and 3, respectively, while using 13 mt genes) will yield similar topology and resolution—and this will be tested in upcoming studies.

By inspecting our alignments, we detected at least one instance of convergent switching between synonymous Serine codon families similar to the findings of Rota-Stabelli et al. (2013) and conducted an amino acid analysis to test for the effects of this phenomenon (not shown). This analysis yielded a near-identical topology and branch support as the nucleotide analyses, with the exception of the *Pseudomuriella* + *Mychonastes* clade, which lost support and became a grade instead. These problematic amino acid positions were few and do not impact our overall phylogenetic conclusions.

Combining the mt data with the nuclear and chloroplast data resulted in a topology identical to that based on mt data only (fig. 5 and supplementary fig. S10, Supplementary Material online). Although most nodes received equal or increased support in the combined analyses, the placement of *Chromochloris* was poorly supported compared with the mt phylogeny, indicating a stronger conflict with the chloroplast or nuclear data. We currently are exploring the information content in the three data sets as well as pairwise comparisons of individual genes.

It was suggested in previous studies that gene order in organellar genomes can be used for phylogenetic inference (e.g., Turmel et al. 2013). Our BADGER analysis yielded a tree that was well supported at nodes grouping closely related taxa (e.g., *B. aerius* and *B. minor*), but moderately to poorly supported at deeper nodes, yet importantly did not strongly conflict with phylogenies estimated from sequence data. It is possible that mt genomes do not have enough information (only 36 characters in our case) for this type of analysis. However, with denser taxon sampling gene order data may prove phylogenetically useful in the future.

Because our data set is very taxon-poor, we do not present the mt phylogeny as a final summary of the species relationships within Sphaeropleales. However, we find the high levels of variation in the mt protein-coding genes and the strong support for the presented relationships promising. Mt genes are rarely used in green algal systematics. So far, nuclear rDNA and a handful of chloroplast genes have been used in most cases. The results of our study suggest that the use of mt genes may help inform some of the phylogenetic relationships that thus far have been problematic to resolve, as suggested by Turmel et al. (2013).

## Conclusions

Our study demonstrates great diversity among sphaeroplealean mt genomes. Although gene content is conserved, we find considerable variation in genome size and especially intron content. We present evidence for a convergent arrangement of *rns1* and *rns2* in close proximity, and report the usage of a unique stop codon, UCG. Finally, we show the potential of phylogenetic utility of the mt protein-coding genes. Examination of a sole representative for the order could not reveal this novel information. We hereby make a case for increased taxon sampling in genomic studies in order to address the patterns and processes of organellar genome evolution in green algae.

## Supplementary Material

Supplementary tables S1 and S2 and figures S1–S11 are available at *Genome Biology and Evolution* online (http://www.gbe.oxfordjournals.org/).

Supplementary Data
