# Supplementary material for: Gene Arrangement Convergence, Diverse Intron Content, and Genetic Code Modifications in Mitochondrial Genomes of Sphaeropleales (Chlorophyta)
Source: Genome Biol Evol. 2014 Aug 8;6(8):2170–80. doi: 10.1093/gbe/evu172 (PMC4159012; doi:10.1093/gbe/evu172)
Supplement: Supplementary Data [file supp_6_8_2170__index.html]

Gene arrangement convergence, diverse intron content, and genetic code modifications in mitochondrial genomes of Sphaeropleales (Chlorophyta). — Gene Arrangement Convergence, Diverse Intron Content, and Genetic Code Modifications in Mitochondrial Genomes of Sphaeropleales (Chlorophyta) — Supplementary Data 

# Gene Arrangement Convergence, Diverse Intron Content, and Genetic Code Modifications in Mitochondrial Genomes of Sphaeropleales (Chlorophyta)

## Supplementary Data

files

**Files in this Data Supplement:**

- Supplementary Data - pdf file
- Supplementary Data - pdf file
- Supplementary Data - pdf file
- Supplementary Data - pdf file
- Supplementary Data - pdf file
