# Supplementary material for: Gene Arrangement Convergence, Diverse Intron Content, and Genetic Code Modifications in Mitochondrial Genomes of Sphaeropleales (Chlorophyta)
Source: Genome Biol Evol. 2014 Aug 8;6(8):2170–80. doi: 10.1093/gbe/evu172 (PMC4159012; doi:10.1093/gbe/evu172)
Supplement: Supplementary Data [file supp_evu172_Dispersed_repeats_S13.pdf]

Species	Repeat unit	# occurrences
Chlorotetraedron incus	GTTTTAT	31
	TTGATT	101
	TATTTTT	103
	TTCTTT	106
	TTTTTGA	128
	TTTTGG	360
Chromochloris zofingiensis	TGCTAT	30
	ATTATA	65
	AATTTTA	143
	AATTTA	449
Kirchneriella aperta	CTATTCT	27
	TTCATAT	28
	TTTATAT	44
	TTGTTTT	66
	ATTTAT	101
Neochloris aquatica	TTAATAA	25
	AATATTT	31
	TTAATT	98
Ourococcus multisporus	TTAATTA	58
	TTTTTAG	59
	TTGGTT	106
	TAATTA	136

	TTAATT	183
	TTATTTT	206
	TTTTATT	220
	TTTTTAA	304
	TTTTAT	385
Pseudomuriella schumacherensis	TTTTGG	75

Table S13. Small dispersed repeats in mitochondrial genomes of Sphaeropleales, detected by RepeatMasker (Smit et al. 1996-2010). Only elements longer than 5 bp and repeated more than 10 times within a genome are listed. Colors indicate elements found in more than one species.
