# Supplementary figures and images for: Gene Arrangement Convergence, Diverse Intron Content, and Genetic Code Modifications in Mitochondrial Genomes of Sphaeropleales (Chlorophyta)
Source: Genome Biol Evol. 2014 Aug 8;6(8):2170–80. doi: 10.1093/gbe/evu172 (PMC4159012; doi:10.1093/gbe/evu172)

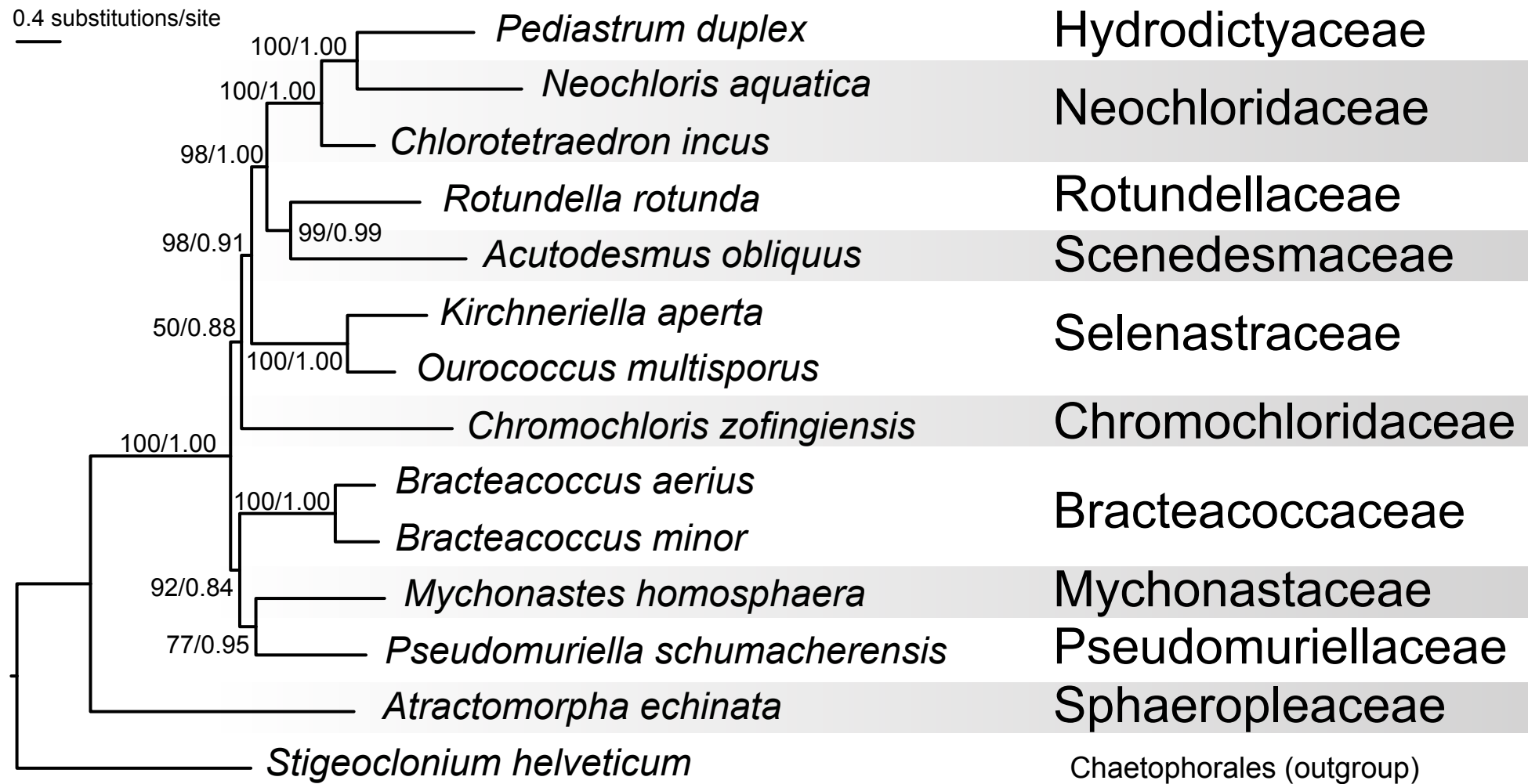

Supplement: Supplementary Data [file supp_evu172_FigS10-alldatatree.pdf]

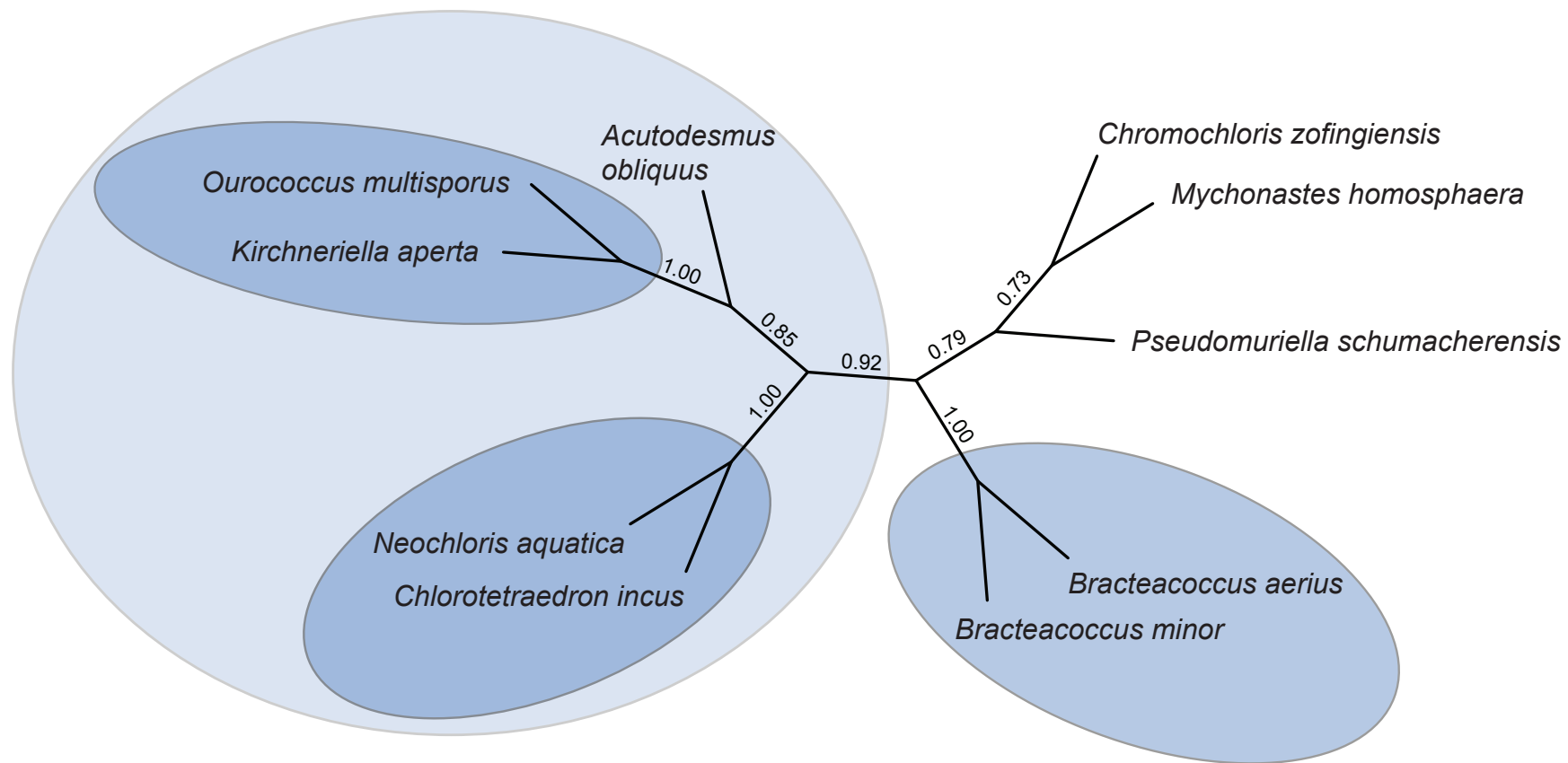

Supplement: Supplementary Data [file supp_evu172_FigS11-badger-color.pdf]

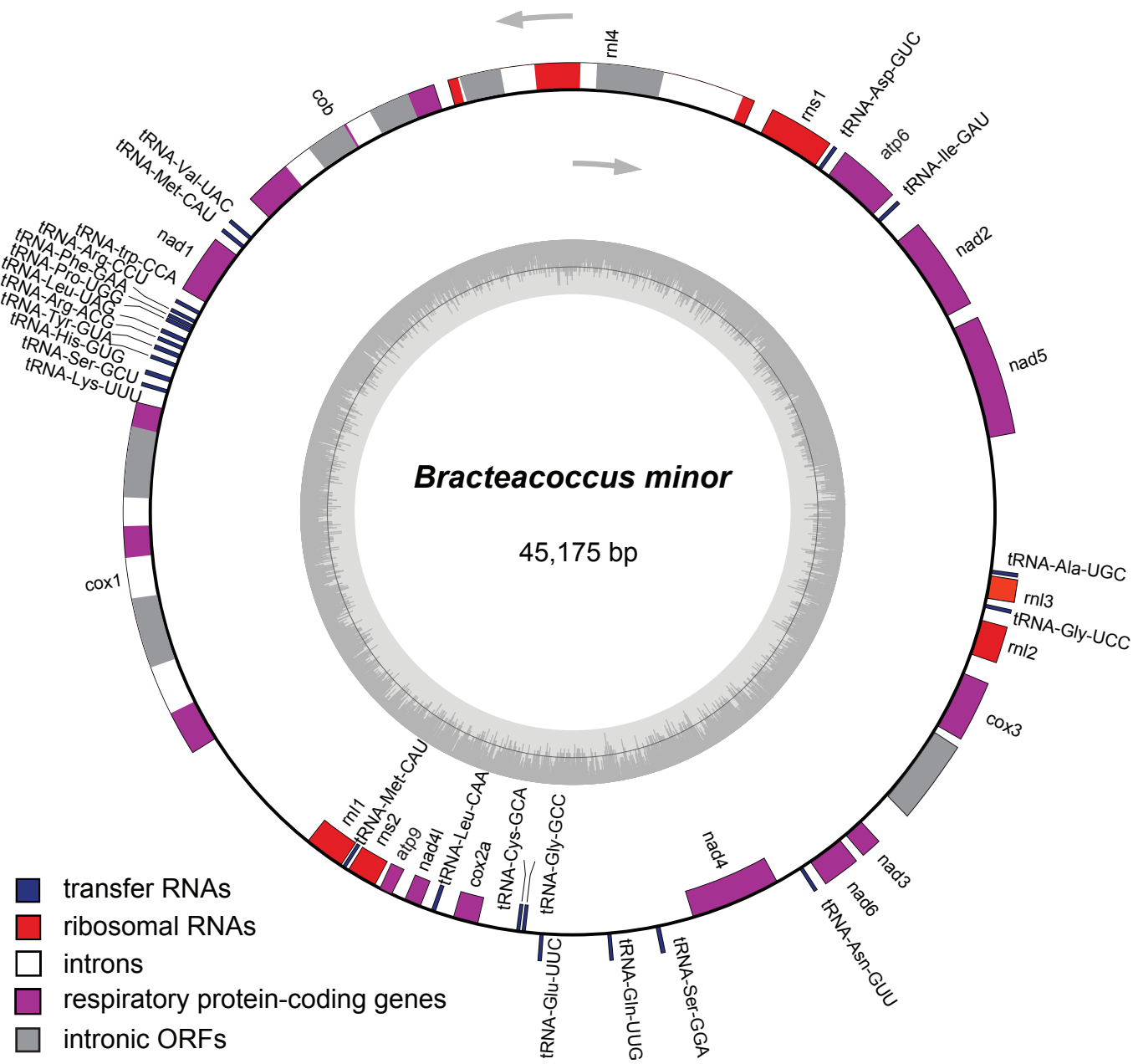

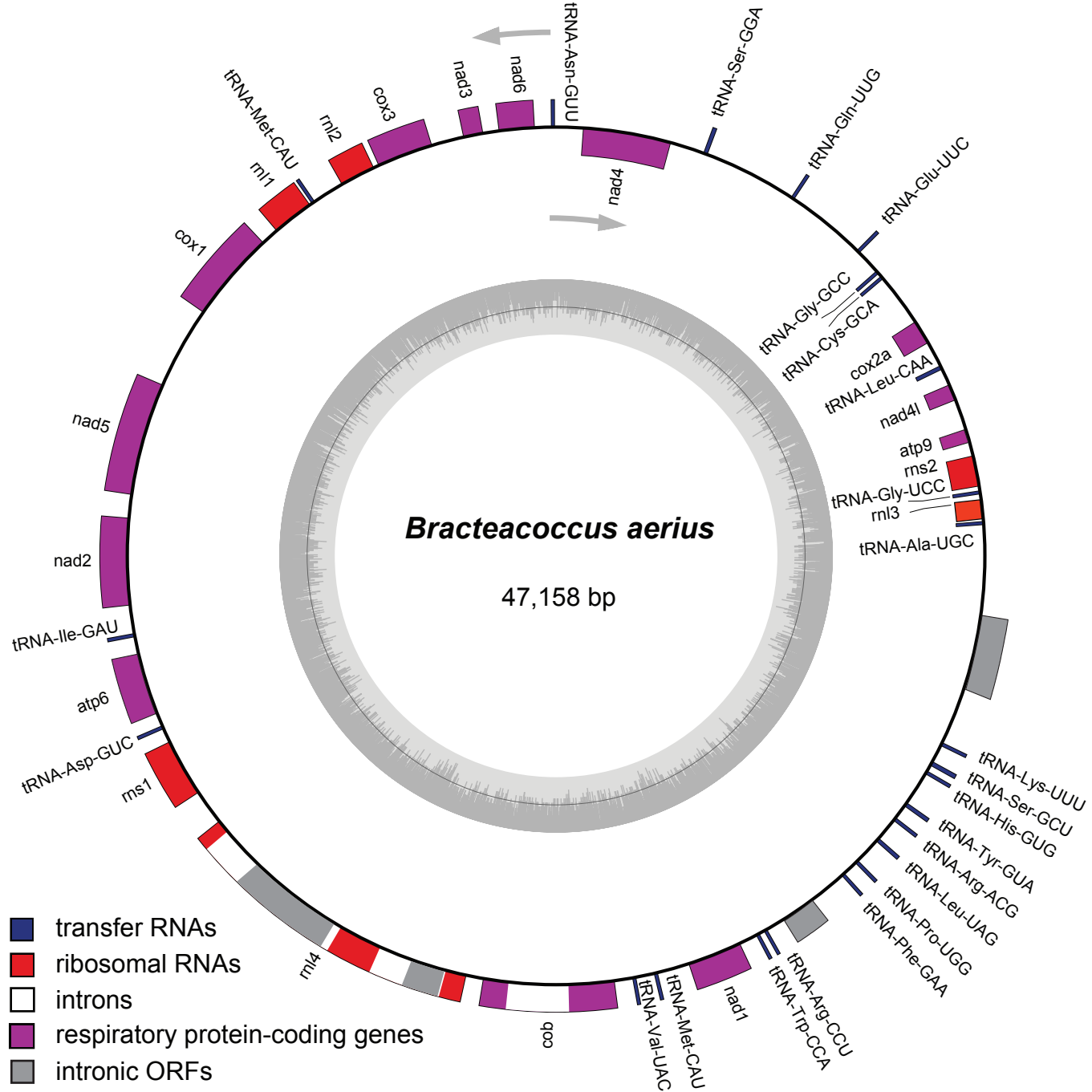

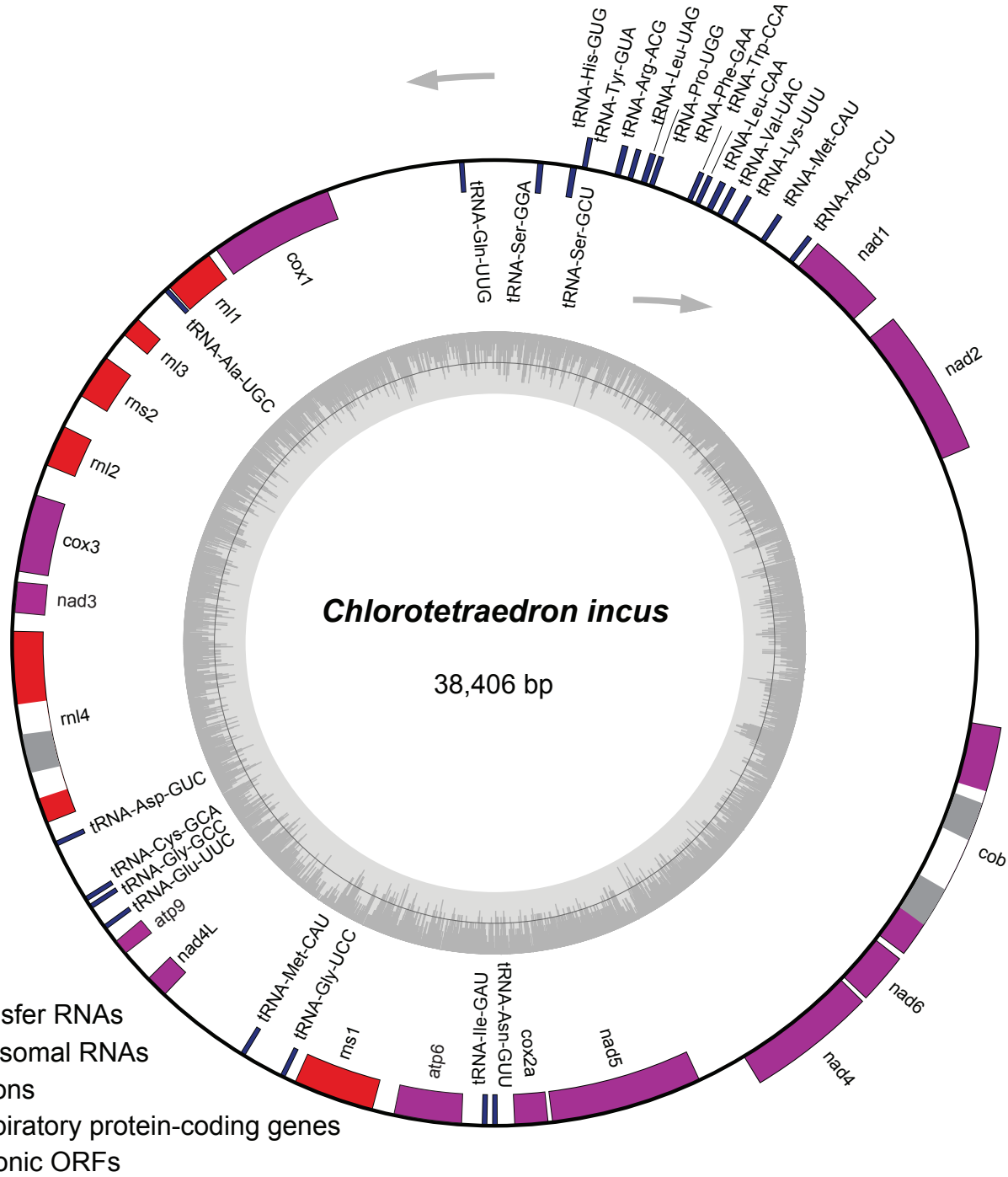

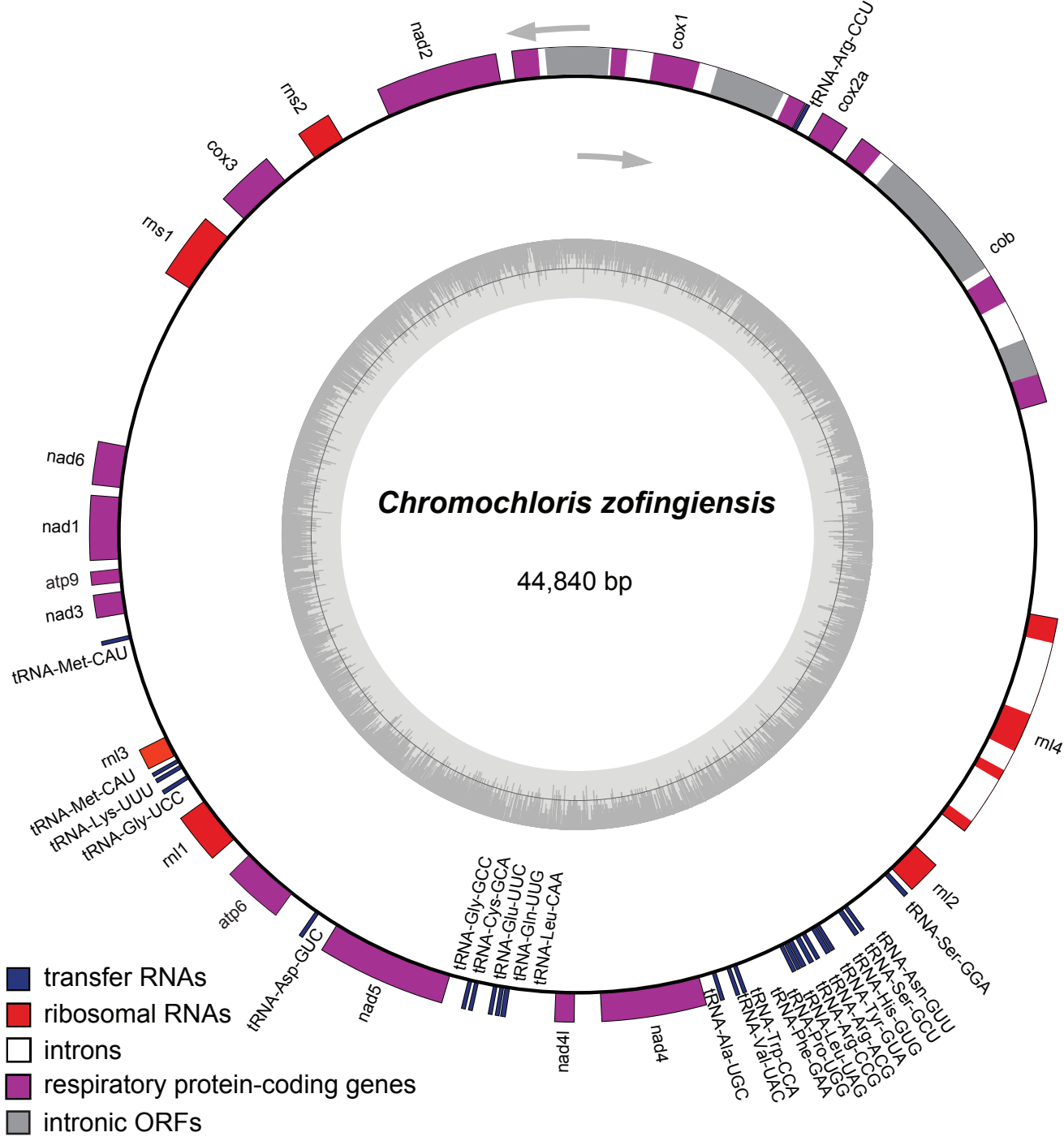

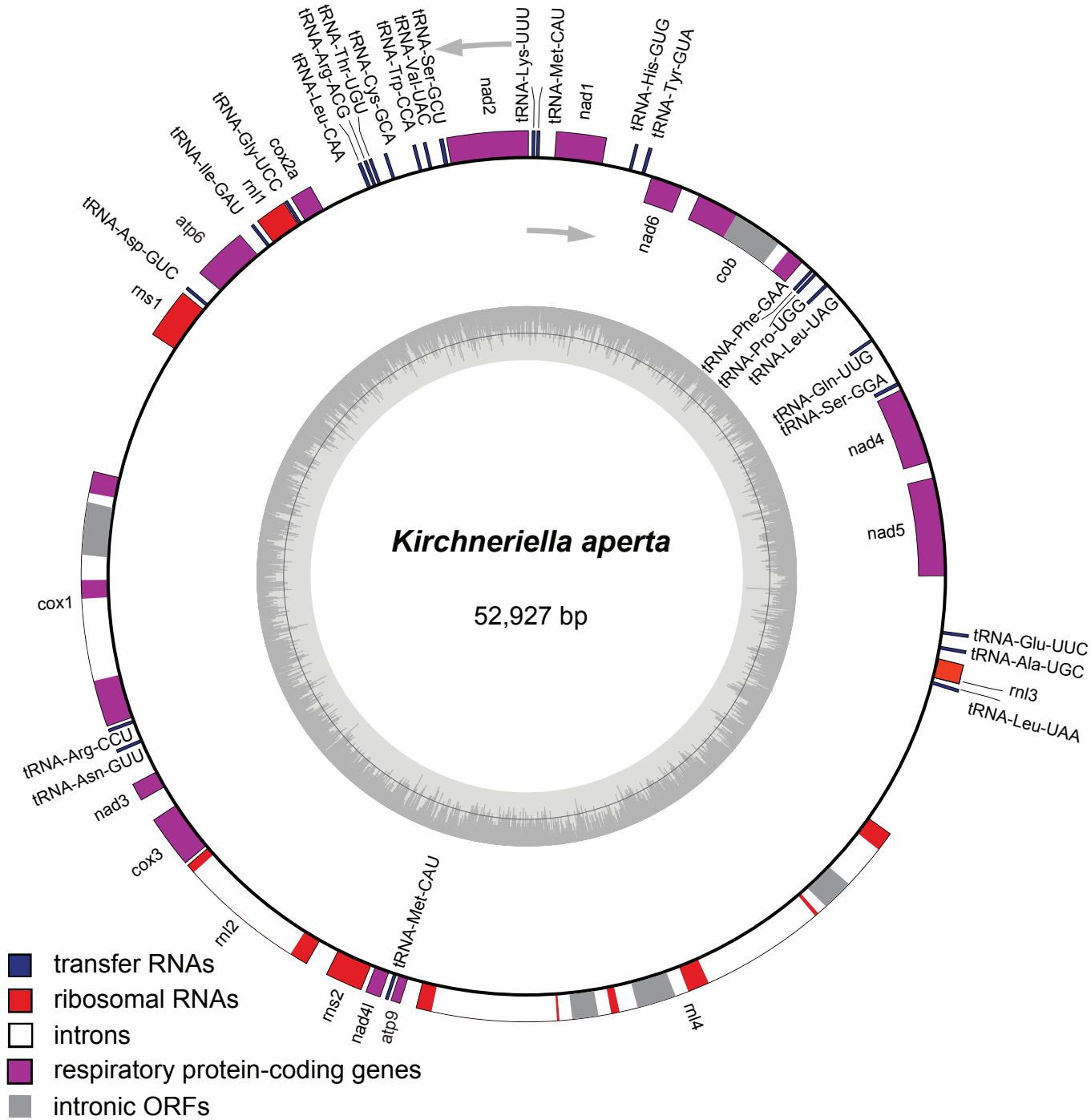

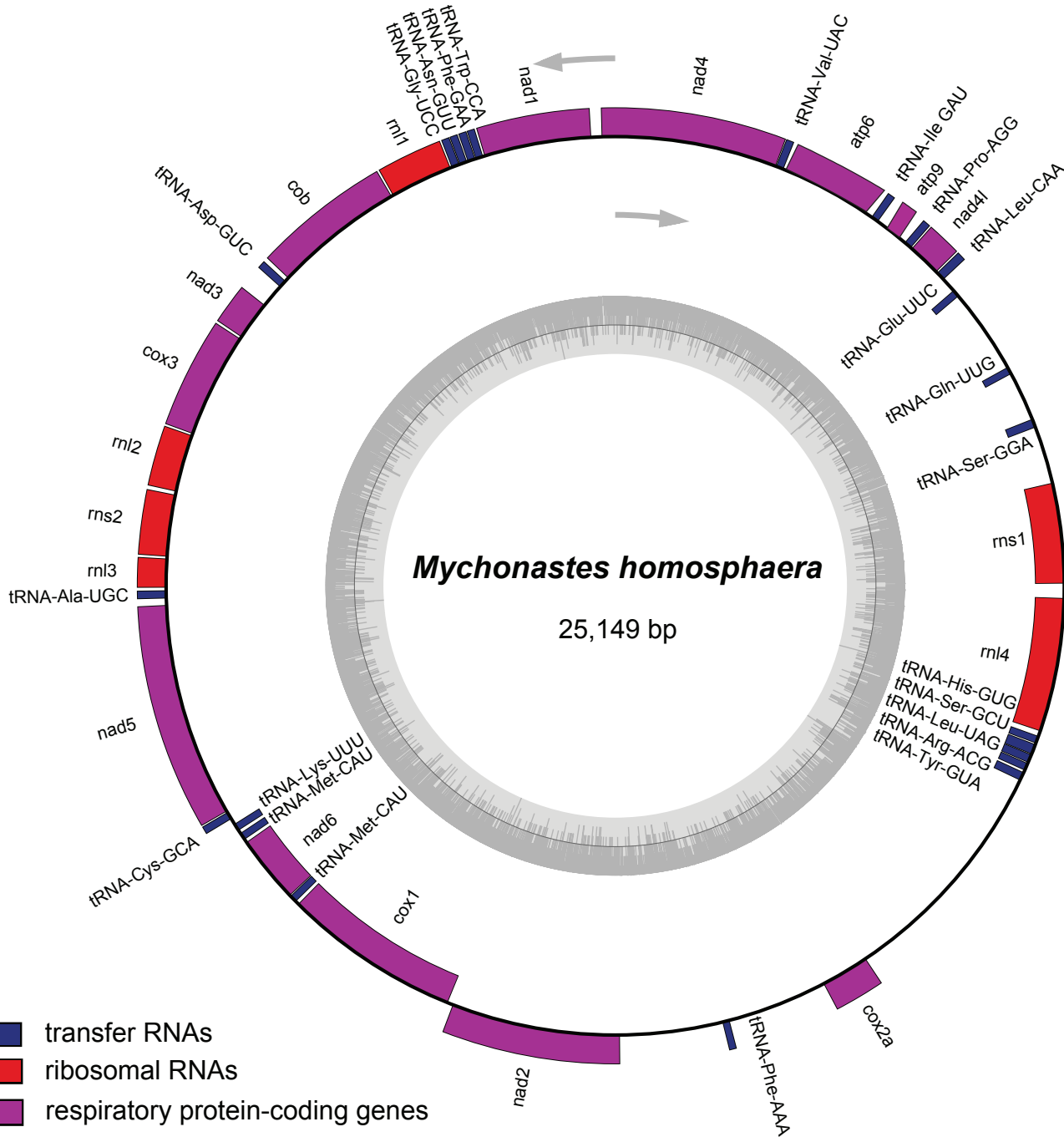

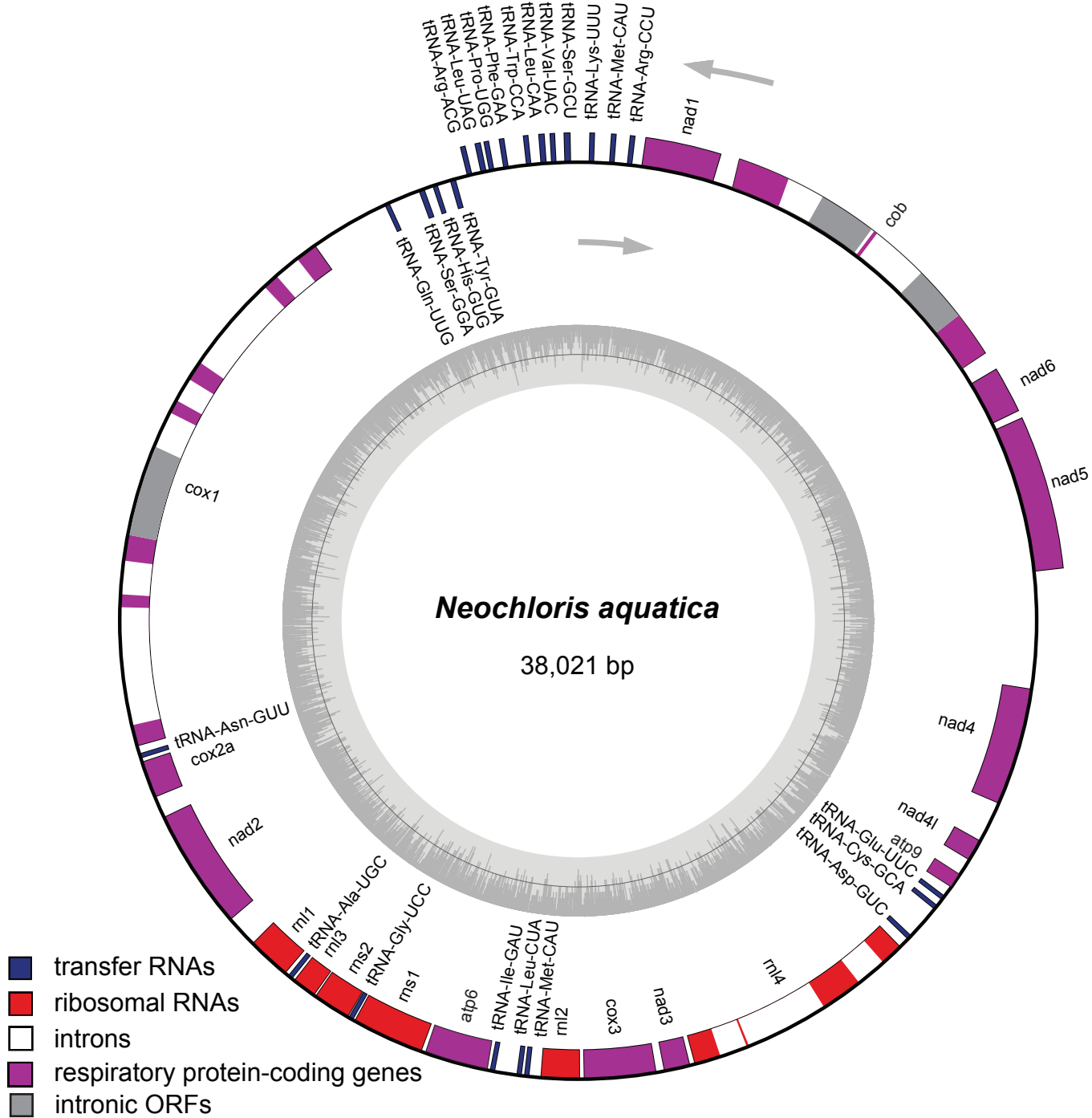

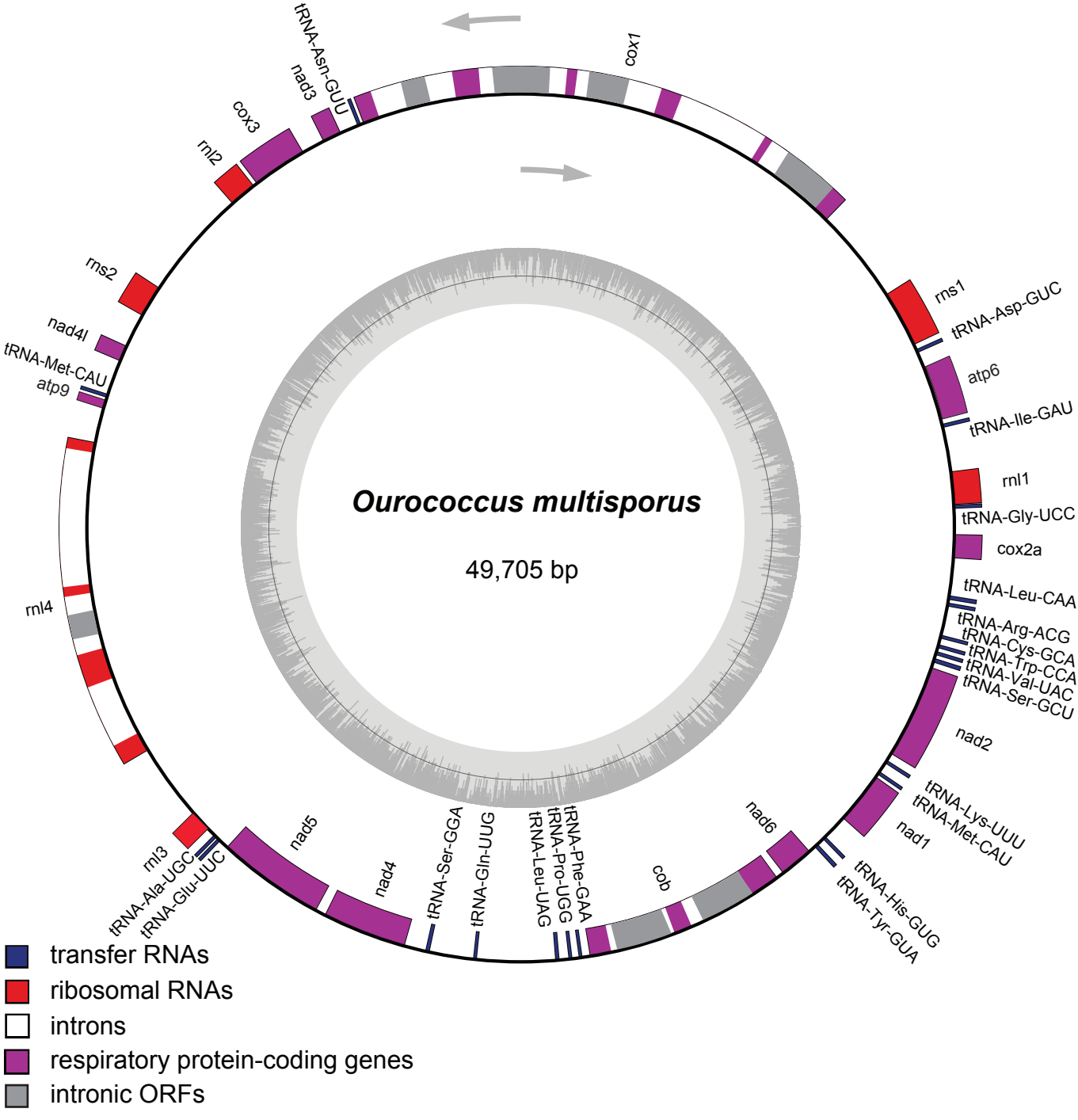

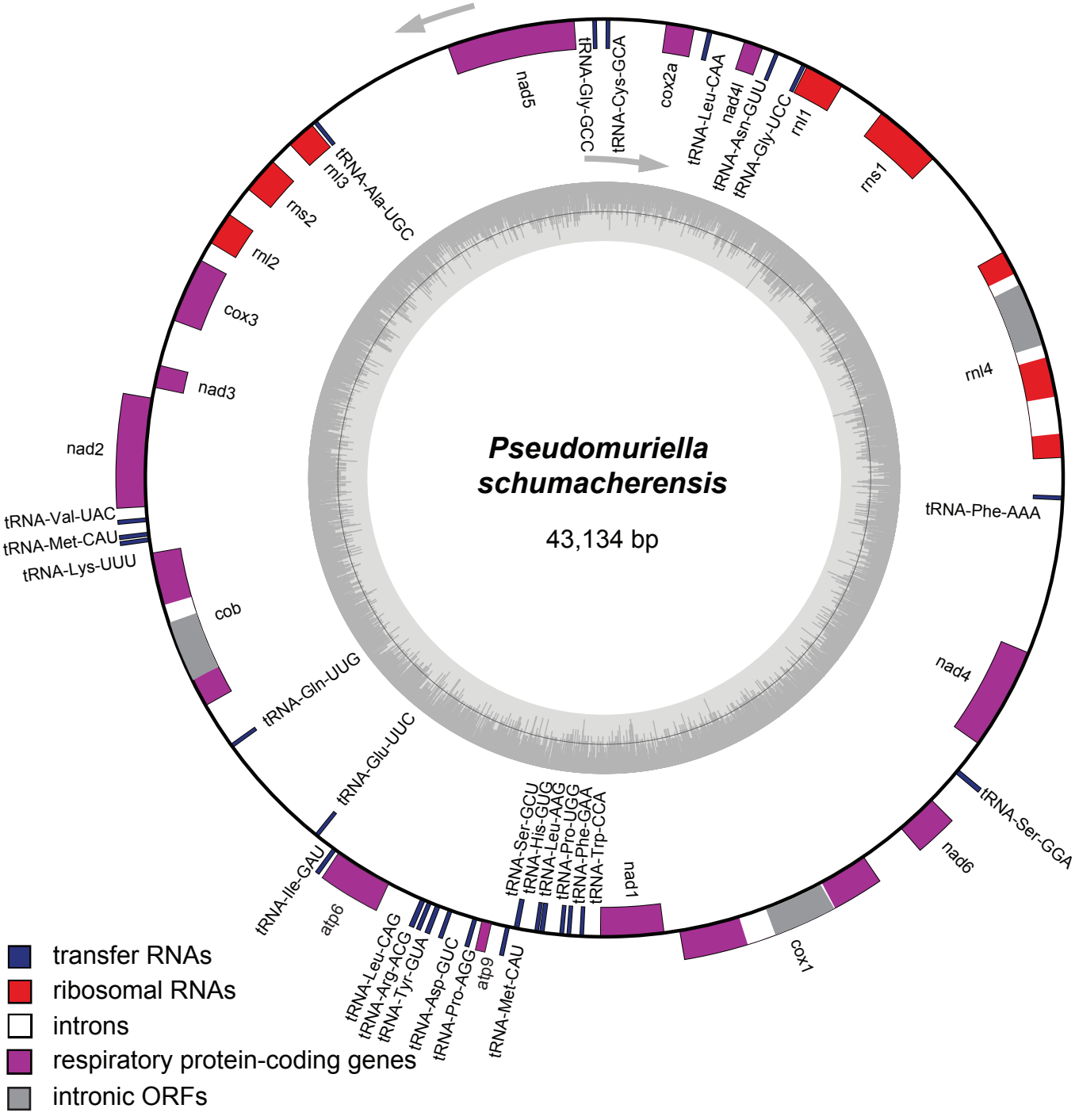

Supplement: Supplementary Data [file supp_evu172_Fucikova_etal_S1-S9.pdf]
