# Supplementary material for: Gene Arrangement Convergence, Diverse Intron Content, and Genetic Code Modifications in Mitochondrial Genomes of Sphaeropleales (Chlorophyta)
Source: Genome Biol Evol. 2014 Aug 8;6(8):2170–80. doi: 10.1093/gbe/evu172 (PMC4159012; doi:10.1093/gbe/evu172)
Supplement: Supplementary Data [file supp_evu172_TableS12_tRNAs.pdf]

tRNA type	Anticodon	*Bracteacoccus aerius*	*Bracteacoccus minor*	*Chlorotetraedron incus*	*Chromochloris zofingiensis*	*Kirchneriella aperta*	*Mychonastes homosphaera*	*Neochloris aquatica*	*Ourococcus multisporus*	*Pseudomuriella schumacherensis*	*Acutodesmus obliquus*	*Pediastrum duplex*	*Rotundella rotunda*	*Atractomorpha echinata*
**Ala**	**UGC**	+	+	+	+	+	+	+	+	+	+	+	+	+
**Arg**	**ACG**	+	+	+	+	+	+	+	+	+	+	+	+	?
	**CCU**	+	+	+	+	+		+			+	?	?	?
	**CCG**				+							?	?	?
	**UCU**										+	?	+	?
**Asn**	**GUU**	+	+	+	+	+	+	+	+	+	+	+	+	?
**Asp**	**GUC**	+	+	+	+	+	+	+	+	+	+	+	+	?
**Cys**	**GCA**	+	+	+	+	+	+	+	+	+	+	+	+	?
**Gln**	**UUG**	+	+	+	+	+	+	+	+	+	+	+	+	?
**Glu**	**UUC**	+	+	+	+	+	+	+	+	+	+	+	+	?
**Gly**	**GCC**	+	+	+	+					+		+	+	?
	**UCC**	+	+	+	+	+	+	+	+	+	+	+	+	+
**His**	**GUG**	+	+	+	+	+	+	+	+	+	+	+	+	+
**Ile**	**GAU**	+	+	+		+	+	+	+	+	+	+	+	?
	**UAU**										+	?	?	?
**Leu**	**UAG**	+	+	+	+	+	+	+	+			+	?	+
	**CAA**	+	+	+	+	+	+	+	+	+	+	+	+	?
	**UAA**					+						?	?	?
	**CUA**							+			+	+	?	?
	**AAG**									+	+	?	+	?
	**CAG**									+	+	?	+	?
**Lys**	**UUU**	+	+	+	+	+	+	+	+	+	+	+	+	?
**Met**	**CAU**	+	+	+	+	+	+	+	+	+	+	+	+	?
	**CAU**	+	+	+	+	+	+	+	+	+	+	+	+	?
**Phe**	**GAA**	+	+	+	+	+	+	+	+	+	+	+	+	+
	**AAA**									+		?	?	?
**Pro**	**UGG**	+	+	+	+	+		+	+	+	+	+	+	+
	**AGG**						+			+		?	?	?
**Ser**	**GCU**	+	+	+	+	+	+	+	+	+	+	+	+	?
	**GGA**	+	+	+	+	+	+	+	+	+	+	?	+	?
	**UGA**													+
**Thr**	**UGU**					+						?	?	?
**Tyr**	**GUA**	+	+	+	+	+	+	+	+	+	+	+	+	?
**Trp**	**CCA**	+	+	+	+	+	+	+	+	+	+	+	+	+
**Val**	**UAC**	+	+	+	+	+	+	+	+	+	+	+	+	?

Table S12. Transfer RNA genes present in the mitochondrial genomes of Sphaeropleales. Question marks (?) indicate unknown presence/absence in partially sequenced genomes. Colors highlight tRNAs corresponding to the UAG codon (standard stop codon, purple) and the UCA codon (stop codon in most Sphaeropleales, blue). *Acutodesmus obliquus* is a synonym of *Scenedesmus obliquus*.
